# Impaired cognitive and motor function are coincident with l-DOPA-induced dyskinesia in a model of Parkinson’s disease

**DOI:** 10.1038/s41598-023-44869-y

**Published:** 2023-10-17

**Authors:** Mariah J. Lelos, Ellen M. Murphy, Hanna S. Lindgren, Stephen B. Dunnett, Emma L. Lane

**Affiliations:** 1 Brain Repair Group, School of Biosciences, Cardiff University, Cardiff, CF10 3AT Wales, UK; 2 School of Pharmacy and Pharmaceutical Sciences, Cardiff University, Cardiff, Wales, UK

**Keywords:** Diseases, Neurological disorders, Neuroscience, Motivation, Sensorimotor processing

## Abstract

Dopamine transmission has been implicated in motor and cognitive function. In Parkinson’s disease (PD), dopamine replacement using the precursor drug l-DOPA is the predominant treatment approach, but long-term exposure leads to the onset of dyskinesias (LIDs). Chronic l-DOPA exposure has been associated with changes in gene expression and altered cortico-striatal plasticity. The aim of this research was to assess the functional consequence of long-term l-DOPA exposure on cognitive and motor function using a rodent model of PD. Across two independent experiments, we assessed the impact of chronic l-DOPA exposure, or a control D_2_R agonist, on motor and cognitive function in intact and in hemi parkinsonian rats, in the absence of drug. Abnormal involuntary movements associated with LID were measured and brain tissues were subsequently harvested for immunohistochemical analysis. Exposure to chronic l-DOPA, but not the D_2_R agonist, impaired motor and cognitive function, when animals were tested in the absence of drug. A meta-analysis of the two experiments allowed further dissociation of l-DOPA -treated rats into those that developed LIDs (dyskinetic) and those that did not develop LIDs (non-dyskinetic). This analysis revealed impaired cognitive and motor performance were evident only in dyskinetic, but not in non-dyskinetic, rats. These data reveal a functional consequence of the altered plasticity associated with LID onset and have implications for understanding symptom progression in the clinic.

## Introduction

Dopamine plays a critical role in both motor and cognitive function. Parkinson’s disease (PD) is primarily characterised by nigrostriatal dopaminergic loss and typified by progressive motor dysfunction. However, PD is now widely recognised as involving cognitive impairment in up to 50% of cases^1^, with a cumulative prevalence of dementia of up to 80%^2^. Dopamine replacement is the predominant treatment approach, using the dopamine precursor l-3,4-dihydroxyphenylalanine (l-DOPA). Although highly effective, with disease progression prolonged l-DOPA use is associated with the generation of abnormal involuntary movements, known as l-DOPA -induced dyskinesias (LIDs). Interestingly, there is growing evidence to suggest that there is a link between the risk of cognitive impairment and the propensity to develop LID, however the precise relationship remains unclear^3,4^. Non-invasive brain stimulation techniques illustrate that l-DOPA itself can cause dose-dependent non-linear effects on inhibitory and facilitatory cortical plasticity, and that established LID are specifically associated with abnormal cortical facilitation^5^. Studies in animal models, predominantly the 6-hydroxydopamine (6-OHDA) lesioned rat, have demonstrated that long term l-DOPA administration, and LID in particular, are associated with a host of changes in striatal cortical gene and protein expression with physiological consequences including altered cortico-striatal plasticity^6,7^. However, the relationship between these physiological changes and a functional impact on cognition has not been explored. We have therefore used a rodent model of dopamine depletion and pulsatile dopamine replacement with l-DOPA to trigger LID, before testing cognitive function in a pretrained automated operant lateralised choice reaction time (LCRT) task^8,9^. Performance on this task, which requires both motor and cognitive processing, can be broken down into selected components, enabling a dissection of the alterations in function affected by the aberrant plasticity associated with LID. In a second experiment, we compared the impact of l-DOPA to Bromocriptine, which is a D2 receptor agonist that is not associated with dyskinesia onset. This was to determine whether long-term functional changes are specific to l-DOPA/LID onset, or whether they also emerge with exposure to dopamine agonists that are also prescribed to PD patients. This approach allows us to directly explore the impact of long-term l-DOPA exposure on motor and cognitive function, which is challenging to assess systematically in a clinical scenario.

## Methods

### Experiment 1

**Figure 1:**
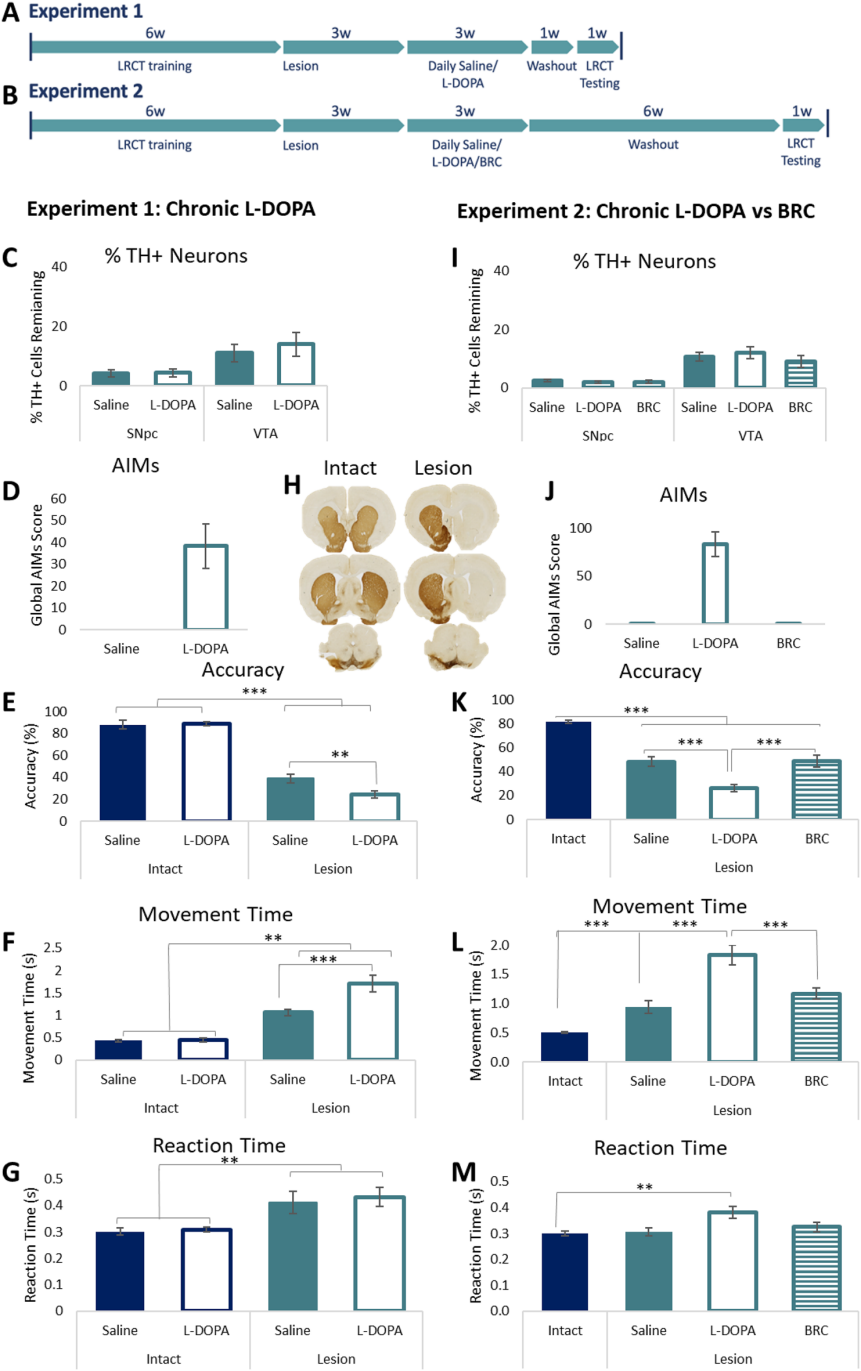
Chronic l-DOPA treatment impairs motor and cognitive function. Timeline of experimental events in Experiments 1 (**A**) and 2 (**B**). (**C**) In Experiment 1, rats were given dopamine depleting lesions of the MFB, resulting in loss of TH + neurons in the substantia nigra pars compacta (SNpc) and ventral tegmental area (VTA). No differences in cell loss were observed between saline and l-DOPA treated groups in either subregion (ps = n.s.) (**D**) Rats exposed to chronic l-DOPA developed AIMS, while rats given saline injections did not develop AIMs. (**H**) Representative histology of an intact and a 6-OHDA MFB lesion rat, show immunostaining for tyrosine hydroxylase (TH) in the striatum and midbrain illustrating the extent of the lesion. Rats were tested in the lateralised choice reaction time (LCRT) task to assess cognitive function (accuracy), motor speed (movement time) and the time to react to the stimulus onset (reaction time). Overall, rats with MFB lesions showed impaired accuracy ((**E**); Group: F_1,40_ = 220.74, p < 0.001), slower motor responses ((**F**); Group: F_1,40_ = 43.19, p < 0.001) and slower reaction times ((**G**); Group: F_1,40_ = 9.82, p < 0.01) than intact rats. Lesioned rats treated with l-DOPA were significantly more impaired cognitively than saline treated rats (Group*Drug: F_1,40_ = 4.13, p < 0.05; Lesion rats, saline vs l-DOPA, p < 0.01) and had slower motor responses (Group*Drug: F_1,40_ = 4.56, p < 0.05; Lesion rats, saline vs l-DOPA, p = 0.001). (**I**) In Experiment 2, no differences in TH + cell loss were evident between saline, l-DOPA or BRC treated rats in either the SNpc (p = n.s.) or the VTA (p = n.s.). (**J**) Rats treated chronically with l-DOPA developed AIMs, while rats treated with saline or the D_2_ agonist BRC did not develop AIMs. The behaviour of intact rats treated with saline, l-DOPA or BRC did not differ on any measure, therefore rats were combined into a single control group. Rats with MFB lesions showed impaired accuracy ((**K**); Group: F_3,81_ = 73.11, P < 0.001; Intact vs all lesion groups, ps < 0.001) and motor responses ((**L**) Group: F_3,81_ = 30.44; Intact vs all lesion groups, ps < 0.001). After chronic drug treatment, only l-DOPA treated rats showed further impairment in accuracy ((**K**); l-DOPA vs Saline/BRC, ps < 0.001), motor response ((**L**); l-DOPA vs Saline/BRC, ps < 0.001) and reaction time ((**M**); Group: F_3,81_ = 4.17, p < 0.01; Intact vs l-DOPA, p < 0.01). In contrast, treatment with BRC had no impact on any aspect of task performance relative to saline control. *p < 0.05, **p < 0.01, ***p < 0.001, n.s. = non-significant.

Forty-four rats were trained on the LCRT task^8,9^ for 6 weeks before a subset of 28 rats received 6-OHDA MFB lesions (Fig. 1A). Rats were tested for motor impairments to validate the lesion efficiency. All rats showed spontaneous rotational behaviour and demonstrated paw-use bias on the cylinder test post-lesion. No differences in behaviour were evident between the subsequent groups (*Rotations*. Saline: 39.5 ± 4.2, l-DOPA: 39.1 ± 4.0; *Cylinder Bias*. Saline: 78.8% ± 8.4, l-DOPA: 72.4% ± 8.1).

Rats received daily injections of either saline (intact n = 8, lesion n = 13) or l-DOPA (intact n = 8, lesion n = 15) for 3 weeks and abnormal involuntary movements (AIMs) were scored^10^. After a 1-week drug washout period, rats were retested on the LCRT task for 5 days in the absence of drug.

### Experiment 2

Eight-five rats were trained on the LCRT task for 6 weeks before the 6-OHDA MFB (n = 55; Fig. 1B). Lesion validity was assessed post-lesion as in Experiment 1. No differences in behaviour were evident between the subsequent groups (*Rotations*. Saline: 41.5 ± 5.1, l-DOPA: 36.2 ± 5.9, Bromocriptine (BRC): 39.8 ± 5.4; *Cylinder Bias*. Saline: 79.8% ± 6.7, l-DOPA: 71.8% ± 4.5, BRC: 71.9% ± 4.8). Daily injections of saline (intact n = 10, lesion n = 15), l-DOPA (intact n = 10, lesion n = 25), or BRC (intact n = 10, lesion n = 15) were given for three weeks and AIMS scored. After a 6-week washout period, rats were tested on the LCRT task for 5 days.

### Animals

Female lister hooded rats (200–225 g; Charles River, UK) were housed in groups of four per cage with ad libitum access to water, and food intake was restricted to maintain 85–90% of their free feeding weight during testing. All experiments were approved by Cardiff University Animal Welfare and Ethics Board (AWERB; ethics committee) and were conducted in accordance with the UK Animals (Scientific Procedures) Act 1986, under UK Home Office Licence PPL 30/3036. All procedures were conducted in line with the ARRIVE guidelines.

### Lateralised choice reaction time task (LCRT)

The LCRT has been used previously in the unilateral 6-OHDA model^8,9^. The LCRT is conducted in 9-hole operant box apparatus (Paul Fray, U.K.), which were constructed of aluminium (25 × 25 cm) with a grid floor, and the back wall housed an array of nine holes, each of which contained a light-emitting diode to provide a visual stimulus, and a vertical infrared beam with a photocell detector, which detected nose poke responses into the hole. A food magazine in the middle of the opposite wall signalled the delivery of 45-mg sucrose reward pellets (TestDiet, IN, USA). On-line data collection was controlled by BNC software (Campden Instruments, UK). Pre-training on the LCRT was conducted over an 8 week period. The central location in the array and a lateral “response” location (one space away, left of the centre or right of the centre) were utilized. Rats were trained to hold their noses in the central location for a variable delay to initiate brief presentation (200 ms) of a lateralised light. A correct response into the lateralised hole triggered delivery of a sucrose pellet into the magazine (45 mg, TestDiet, IN, USA). Errors were recorded and resulted in a 2 s time out. Failure to maintain hold in the central location for the variable delay duration resulted in a time out and an unusable trial. The session duration was 30 min. ‘Accuracy’ measures the ability of the rat to correctly localise a response in the correct, rather than the incorrect, nosepoke operandum. ‘Reaction time’ refers to the time it takes for the rat to remove its nose from the centre hole after the stimulus light flashes. This measure incorporates elements of both attentional processing and motor function. ‘Movement time’ refers to the time it takes from removing the animal’s nose from the centre hole to reaching the correct nocepoke operandum.

### Lesion surgery and histology

6-OHDA lesions were performed as described previously^9^, and culled via transcardiac perfusion using pentobarbital, phosphate-buffered saline as a pre-wash and 4% paraformaldehyde as a fixative. Immunohistochemical analysis of tyrosine hydroxylase (TH) positive neurons in the substantia nigra was conducted with primary anti-TH antibody (MAB318; 1:2000, Millipore, UK).

### Drugs

l-DOPA methyl ester (10 mg/kg) was administered in 0.9% saline s.c. with 15 mg/kg benserazide HCl, at 1 ml/kg. BRC was administered i.p. at 2.5 mg/kg (2 ml/kg) (solubilised as described in^11^). All drugs were obtained from Sigma Aldrich, UK. Duration and severity of AIMs were evaluated to obtain a global AIMS score as described in^10^. In the meta-analysis we defined “non-dyskinetic” rats as those achieving an individual element score (amplitude × duration) of 1 or less at any timepoint, while any rat exceeding 1 in any given element was allocated as “dyskinetic”.

### Statistics

Data were analysed via ANOVA using SPSS (IBM, v27) with Group (Intact, Lesion) and Drug (Saline, l-DOPA) as factors for Experiment 1 and Group as the factor for Experiment 2. For the final meta-analysis, Group (Lesion, Dyskinetic, Non-Dyskinetic) were the ANOVA factors. Post-hoc comparisons were conducted using the Sidak correction for multiple comparisons.

## Results

### Chronic l-DOPA impairs cognitive and motor function

Following chronic exposure to l-DOPA, rats with near complete unilateral depletion of midbrain TH + neurons (Fig. 1C,H) developed l-DOPA-induced AIMs (Fig. 1D). On the LCRT test, lesioned rats treated with saline demonstrated impairments in cognitive function (accuracy; Fig. 1E), the motor speed of response to a stimulus (movement time; Fig. 1F) and reaction time, which incorporates elements of motor and attentional function (Fig. 1G). Lesioned rats that had been treated chronically with l-DOPA and then given a 1 week washout period, presented with greater impairment in cognitive function and slower motor responses than their saline injected counterparts (Fig. 1E,F).

### Chronic l-DOPA impairs cognitive and motor function, while BRC has no functional impact

In a second experiment, we again observed the development of l-DOPA induced AIMs after chronic treatment with l-DOPA in rats with near complete loss of TH + neurons in the midbrain (Fig. 1I,J), while rats treated with the dopamine D_2_ receptor agonist BRC showed no abnormal motor manifestations (Fig. 1J). Even after a longer washout period of 6 weeks, the ability of chronic l-DOPA to impair cognitive function (accuracy, Fig. 1K) and motor function (movement and reaction times; Fig. 1L,M) was evident. In contrast, BRC treated rats did not differ to saline treated controls on any measure of cognitive or motor function (Fig. 1K–M).

### The development of cognitive and motor deficits is specific to dyskinetic rats

**Figure 2:**
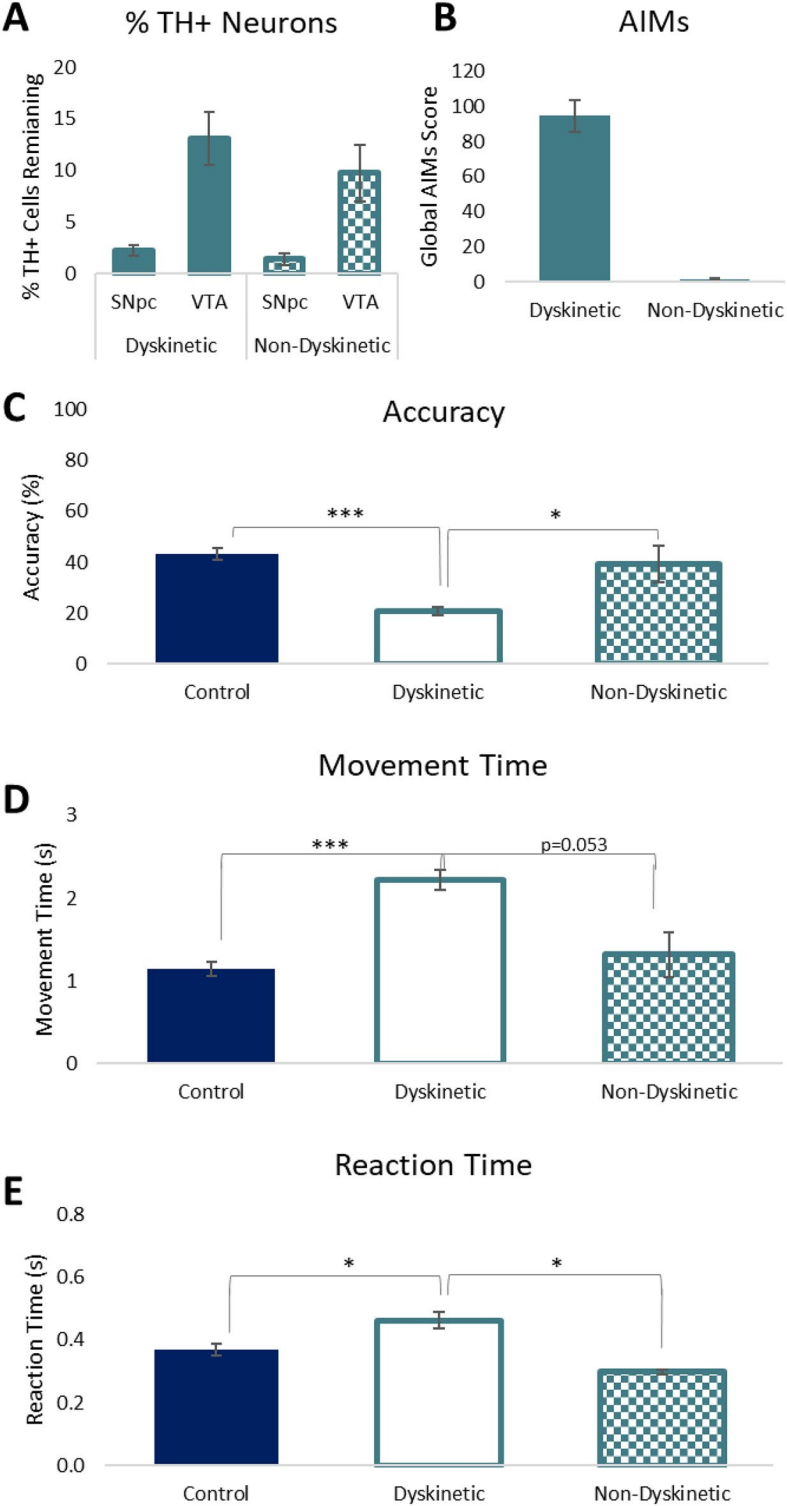
Cognitive and motor dysfunction are associated with the onset of l-DOPA induced dyskinesia. Data from Experiments 1 and 2 were combined and a meta-analysis was conducted, to dissociate the impact of dyskinesia development on behaviour. Subgroups of non-dyskinetic rats were identified from each experiment (n = 6 from Experiment 1, n = 7 from Experiment 2; n = 13 non-dyskinetic rats in total). (**A**) Re-analysis of the histological data revealed no differences in the percentage of TH + neurons in either the SNpc or the VTA (ps = n.s.) between non-dyskinetic and dyskinetic rats. (**B**) AIMs were present in dyskinetic rats, while few or no AIMs were identified in the non-dyskinetic cohort. Dyskinetic rats presented with greater cognitive dysfunction ((**C**); Group: F_2,90_ = 24.80, p < 0.001; Dyskinetic vs Control/Non-dyskinetic, ps < 0.05), slower motor responses ((**D**); Group: F_2,90_ = 19.23, p < 0.001; Dyskinetic vs control, p < 0.05; Dyskinetic vs non-dyskinetic, p = 0.053) and slower reaction times ((**E**); Group: F_2,90_ = 6.89, p < 0.01; Dyskinetic vs Control/Non-dyskinetic, ps < 0.05) than non-dyskinetic, l-DOPA treated rats. *p < 0.05, **p < 0.01, ***p < 0.001, n.s. = non-significant.

During the analysis we observed that a small proportion of the l-DOPA treated rats did not develop overt dyskinesia (6 of 15 and 7 of 25). Therefore, in a subsequent meta-analysis, we separated l-DOPA treated rats into 2 subgroups; those with overt dyskinesia and those without (termed non-dyskinetic) (Fig. 2B). Re-analysis of the histological data revealed no differences in the percentage of TH + cell loss in the SNpc or VTA between dyskinetic and non-dyskinetic groups (Fig. 2A). The performance of these two groups of rats was then assessed in the LCRT task by re-analysis of the combined data from saline and l-DOPA treated rats. The results reveal the presence of LID as a key contributor to the development of cognitive and motor impairments, with significantly worse cognitive function, motor function and reaction time in dyskinetic rats, as compared to non-dyskinetic, l-DOPA treated rats (Fig. 2C–E).

## Discussion

Midbrain dopaminergic depletion produces deficits in both motor and cognitive function. Here we have demonstrated, across two studies, that long-term l-DOPA exposure further impaired motor and cognitive behaviour in a model of PD. This effect was observed in the absence of the drug itself and not observed in rats treated long-term with the D_2_ receptor agonist, BRC used as a comparator in Experiment 2 to control for the additional motor behaviours consistent with dopaminergic receptor stimulation. BRC evokes a rotational response in hemi-lesioned rats but with minimal induction of AIMs-like behaviours and we observed no long-term impact of exposure to BRC on motor or cognitive function. Importantly, despite little variation in dopaminergic degeneration across the two cohorts, a small number of animals in each experiment failed to develop LIDs, a phenomenon that has been associated with the “plasticity potential” of the brain^12^. As a result, it was only by conducting a meta-analysis of the aggregated data that we able to extract and compare the dyskinetic and non-dyskinetic animals, which ultimately identified the onset of LID as the key determinate of behavioural impairment.

The 6-OHDA model represents advanced PD, with extensive nigrostriatal loss and involvement of ventral tegmental pathways. By using the LCRT task, we were able to demonstrate that LID has an additive effect on the dopamine depletion-induced deficits on cognitive function (as measured by accuracy), attentional processing (as measured by reaction time) and movement time. The lesion induced deficits in the task are well established in respect of the dopaminergic lesion and we can be confident that the behavioural consequences of the lesion are directly associated with the dopaminergic loss, supported by the work of Lindgren et al. who demonstrate that overt damage to 5-HT and NA projections had minimal impact on performance in the task^13^. The nature of the cognitive deficit (i.e. accuracy of response) has been explored across a number of experiments. In the earliest studies, the effect of DA depletion in this lateralised task was assessed and the scientists concluded that the response deficit was not driven by sensory neglect, nor sensory inattention. Rather, they speculated that it reflected an ‘output neglect’ or an impairment in response initiation^14,15^. Since then, it was shown that restricting responding to the contralateral side of the array could result in a profound improvement in responding to the nearer of two response holes, while a notable deficit manifested in response to the location that was further away. It was suggested, therefore, that lesioned rats presented with an impairment in encoding responses in egocentric space, which may have manifested due to a distortion in the representation of response space^16^. More recently, it has been suggested that this impairment in spatial encoding may also interact with a motivational dysfunction, potentially influenced by loss of DA along the VTA-nucleus accumbens pathway, which together influence the ability to respond in this lateralised task^8,17^.

LID onset has been associated with several aberrant physiological changes, including the loss of bidirectional synaptic plasticity at corticostriatal synapses, hyperphosphorylation of DARPP-32 and reduced synaptic D_1_/NMDA receptor complexes^18–24^. Other laboratories have reported that the absence of LID in some rodents is associated with fewer alterations in pre- and post-synaptic plasticity, or other neuronal, inflammatory or vascular changes, despite the functional efficacy of l-DOPA being evident in these rats (i.e. l-DOPA evoking a rotational response or improving paw use)^25^. This suggests that the changes in neuronal integrity induced by LID onset may also underpin the long-term cognitive impairment, which implicates a similar underlying corticostriatal synapse. The ability to control for the extent of dopaminergic depletion and l-DOPA exposure, has allowed us to isolate LID development as the sole differentiating factor, which is very challenging to achieve in clinical studies due to the prolonged nature of the effects of l-DOPA. Thus, this study provides a unique insight into the impact of LID onset on cognitive and motor function.

These data support clinical findings that suggest common cortical pathways may under the development of dyskinesia and cognitive decline. In a retrospective cohort study, Yoo and colleagues identified significantly impaired frontal executive function and global cognitive function in Parkinson’s patients that developed LID within 5 years, compared to those without LID, despite no differences between the groups at baseline^4^. In another recent study, impaired executive and attentional function at baseline predicted LID onset^3^. It remains challenging to assess the direct impact of l-DOPA exposure or LID onset in clinical cohorts due to the inherent variability in the patient populations, as well as the inability to control meaningfully for l-DOPA exposure or dose and the extent of disease. Thus, using a rodent model of PD has allowed us to systematically dissociate the impact of l-DOPA or D_2_ agonist exposure on motor and cognitive function in both dyskinetic and non-dyskinetic rats. These data reveal a specific functional consequence of the aberrant plasticity changes associated with LID onset and they have implications for understanding cognitive decline in people with PD.

## Data Availability

The data that support the findings of this study are available from the corresponding author upon reasonable request.

## References

[1] Goldman JG, Litvan I (2011). Mild cognitive impairment in Parkinson’s disease. Minerva Med..

[2] Aarsland D, Kurz MW (2010). The epidemiology of dementia associated with Parkinson’s disease. Brain Pathol..

[3] Luca A, Monastero R, Baschi R, Cicero CE, Mostile G, Davì M, Restivo V, Zappia M, Nicoletti A (2021). Cognitive impairment and levodopa induced dyskinesia in Parkinson’s disease: A longitudinal study from the PACOS cohort. Sci. Rep..

[4] Yoo HS, Chung SJ, Lee YH, Lee HS, Ye BS, Sohn YH, Lee PH (2019). Levodopa-induced dyskinesia is closely linked to progression of frontal dysfunction in PD. Neurology.

[5] Guerra A, Suppa A, D’Onofrio V, Di Stasio F, Asci F, Fabbrini G, Berardelli A (2019). Abnormal cortical facilitation and l-dopa-induced dyskinesia in Parkinson’s disease. Brain Stimul..

[6] Picconi B, Centonze D, Håkansson K, Bernardi G, Greengard P, Fisone G, Cenci MA, Calabresi P (2003). Loss of bidirectional striatal synaptic plasticity in l-DOPA-induced dyskinesia. Nat. Neurosci..

[7] Bove F, Calabresi P (2022). Plasticity, genetics, and epigenetics in l-DOPA-induced dyskinesias. Handb. Clin. Neurol..

[8] Lelos MJ, Dowd E, Dunnett SB (2012). Nigral grafts in animal models of Parkinson’s disease. Is recovery beyond motor function possible?. Prog. Brain Res..

[9] Lelos MJ, Morgan RJ, Kelly CM, Torres EM, Rosser AE, Dunnett SB (2016). Amelioration of non-motor dysfunctions after transplantation of human dopamine neurons in a model of Parkinson's disease. Exp. Neurol..

[10] Breger LS, Dunnett SB, Lane EL (2013). Comparison of rating scales used to evaluate l-DOPA-induced dyskinesia in the 6-OHDA lesioned rat. Neurobiol. Dis..

[11] Westin JE, Andersson M, Lundblad M, Cenci MA (2001). Persistent changes in striatal gene expression induced by long-term l-DOPA treatment in a rat model of Parkinson’s disease. Eur. J. Neurosci..

[12] Linazasoro G (2005). New ideas on the origin of l-dopa-induced dyskinesias: Age, genes and neural plasticity. Trends Pharmacol. Sci..

[13] Lindgren HS, Demirbugen M, Bergqvist F, Lane EL, Dunnett SB (2014). The effect of additional noradrenergic and serotonergic depletion on a lateralised choice reaction time task in rats with nigral 6-OHDA lesions. Exp. Neurol..

[14] Carli M, Jones GH, Robbins TW (1989). Effects of unilateral dorsal and ventral striatal dopamine depletion on visual neglect in the rat: A neural and behavioural analysis. Neuroscience.

[15] Carli M, Evenden JL, Robbins TW (1985). Depletion of unilateral striatal dopamine impairs initiation of contralateral actions and not sensory attention. Nature.

[16] Brown VJ, Robbins TW (1989). Deficits in response space following unilateral striatal dopamine depletion in the rat. J. Neurosci..

[17] Lelos MJ, Harrison DJ, Dunnett SB (2012). Intrastriatal excitotoxic lesion or dopamine depletion of the neostriatum differentially impairs response execution in extrapersonal space. Eur. J. Neurosci..

[18] Lundblad M, Picconi B, Lindgren H, Cenci MA (2004). A model of l-DOPA-induced dyskinesia in 6-hydroxydopamine lesioned mice: Relation to motor and cellular parameters of nigrostriatal function. Neurobiol. Dis..

[19] Cenci MA, Konradi C (2010). Maladaptive striatal plasticity in l-DOPA-induced dyskinesia. Prog. Brain Res..

[20] Picconi B, De Leonibus E, Calabresi P (2018). Synaptic plasticity and levodopa-induced dyskinesia: Electrophysiological and structural abnormalities. J. Neural Transm. (Vienna).

[21] Ghiglieri V, Bagetta V, Pendolino V, Picconi B, Calabresi P (2012). Corticostriatal plastic changes in experimental l-DOPA-induced dyskinesia. Parkinsons Dis..

[22] Fiorentini C, Rizzetti MC, Busi C, Bontempi S, Collo G, Spano P, Missale C (2006). Loss of synaptic D1 dopamine/N-methyl-d-aspartate glutamate receptor complexes in l-DOPA-induced dyskinesia in the rat. Mol. Pharmacol..

[23] Gardoni F, Picconi B, Ghiglieri V, Polli F, Bagetta V, Bernardi G, Cattabeni F, Di Luca M, Calabresi P (2006). A critical interaction between NR2B and MAGUK in l-DOPA induced dyskinesia. J. Neurosci..

[24] Cenci MA, Ohlin KE, Rylander D (2009). Plastic effects of l-DOPA treatment in the basal ganglia and their relevance to the development of dyskinesia. Parkinsonism Relat. Disord..

[25] Konradi C, Westin JE, Carta M, Eaton ME, Kuter K, Dekundy A, Lundblad M, Cenci MA (2004). Transcriptome analysis in a rat model of l-DOPA-induced dyskinesia. Neurobiol. Dis..

